# Das *Lehrnetzwerk Migration und Gesundheit*: Aus- und Weiterbildung konsolidieren und weiterentwickeln

**DOI:** 10.1007/s00103-023-03765-6

**Published:** 2023-09-22

**Authors:** Amand Führer, Stephanie Taché, Henna Riemenschneider, Kayvan Bozorgmehr, Sonia Diaz-Monsalve, Michael Knipper, Claudia Mews, Eva-Maria Schwienhorst-Stich, Ute Siebert, Kai-Uwe Strelow, Sandra Ziegler

**Affiliations:** 1 Institut für Medizinische Epidemiologie, Biometrie und Informatik, Profilzentrum Gesundheitswissenschaften, Martin-Luther-Universität Halle-Wittenberg, Halle (Saale), Deutschland; 2 Bereich Allgemeinmedizin, Medizinische Fakultät Carl Gustav Carus an der Technischen Universität Dresden, Fetscherstr. 74, 01307 Dresden, Deutschland; 3 Sektion Health Equity Studies & Migration, Abteilung Allgemeinmedizin und Versorgungsforschung, Universitätsklinikum Heidelberg, Heidelberg, Deutschland; 4 AG Bevölkerungsmedizin und Versorgungsforschung, Fakultät für Gesundheitswissenschaften, Universität Bielefeld, Bielefeld, Deutschland; 5 Zentrum für Medizin und Gesellschaft, Albert-Ludwigs-Universität Freiburg, Freiburg, Deutschland; 6 Professur für Global Health, Migration und Kulturwissenschaften in der Medizin, Institut für Geschichte, Theorie und Ethik der Medizin, Justus-Liebig-Universität Gießen, Gießen, Deutschland; 7 Institut und Poliklinik für Allgemeinmedizin, Universitätsklinikum Hamburg-Eppendorf, Hamburg, Deutschland; 8 Institut für Allgemeinmedizin und Lehrklinik der Medizinischen Fakultät, Universitätsklinikum Würzburg, Würzburg, Deutschland; 9 Projekt „Empowerment für Diversität – Allianz für Chancengleichheit in der Gesundheitsversorgung“, Charité – Universitätsmedizin Berlin, Berlin, Deutschland; 10 Rudolf Frey Lernklinik, Universitätsmedizin Mainz, Johannes Gutenberg-Universität Mainz, Mainz, Deutschland

**Keywords:** Lehre, Ausbildung, Geflüchtete und Migrant*innen, Diversität, Equity, Teaching, Education, Refugees and Migrants, Diversity, Equity

## Abstract

Patient*innen mit Migrationsgeschichte stoßen im deutschen Gesundheitssystem vielfach auf Zugangsbarrieren, die die Qualität der ihnen zugänglichen Versorgung mindern und ihre Gesundheit beeinträchtigen. Diese Barrieren haben einerseits politische Ursachen, sind jedoch auch auf einen Mangel an migrations- und diversitätsbezogenen Inhalten im Medizinstudium und in anderen gesundheitsbezogenen Studien- und Ausbildungsgängen zurückzuführen. Obwohl die Versorgung von Patientinnen und Patienten mit eigener oder familiärer Migrationsgeschichte zum Alltag gehört, sind dafür relevante Inhalte bislang nicht in den Curricula verankert und werden bestenfalls in Form von Wahlpflichtfächern oder anderen fakultativen Lehrangeboten vermittelt.

Um diese Situation zu verbessern und eine menschenrechtsbasierte, diversitätssensible und Equity-orientierte Weiterentwicklung der Curricula voranzutreiben, hat sich das „Lehrnetzwerk Migration und Gesundheit“ gegründet. Es zielt darauf ab, 1) in der Lehre aktive Personen miteinander zu vernetzen und den Austausch sowie die gemeinsame Weiterentwicklung von Lehrmaterial zu fördern, 2) darauf aufbauend einen Modellkurs „Migration und Gesundheit“ zu entwickeln und 3) Strategien für die longitudinale Implementierung entsprechender Inhalte in Pflichtcurricula zu erarbeiten. Diese Bestrebungen werden von Lehrforschung flankiert. An Mitarbeit im Lehrnetzwerk Interessierte sind herzlich eingeladen, die Autor*innen zu kontaktieren und an diesen Vorhaben mitzuwirken.

## Abstract (en)

Patients with migration history often encounter barriers to accessing healthcare in Germany, which lowers the quality of care available to them and can affect their overall health. These barriers in access to healthcare are due to both adverse health policies and a lack of migration-related – and diversity-sensitive – content in medical and other health profession teaching. Although most healthcare professionals regularly care for patients with individual or generational migration experience in Germany, teaching content relevant to the healthcare of these patients has not yet been anchored in the curriculum. At best, it is taught in the form of electives or other optional courses.

To address this gap, the Teaching Network Migration and Health was created with the goal of promoting the development of human rights-based, diversity-sensitive, and equity-oriented curricula at medical and healthcare professions schools. It aims to (1) connect individuals active in teaching and promote the exchange and collaborative development of teaching materials, (2) use this collective knowledge and experience to develop a model course on migration and health, and (3) develop strategies for the longitudinal implementation of this course into the regular medical and other health professional school curricula. These efforts are flanked by evaluative accompanying research. Anyone interested in joining the network is invited to join and strengthen the network by contacting the authors.

## Einführung

### Der gesellschaftliche Kontext

Migration wird in der deutschen Öffentlichkeit sowie innerhalb des Gesundheitssystems überwiegend anlässlich konkreter und häufig als krisenhaft interpretierter Ereignisse diskutiert. So gab es in den 1990er-Jahren anlässlich der Fluchtbewegungen im Kontext der Balkankriege, Anfang der 2000er-Jahre im Kontext des Berichtes der Süssmuth-Kommission [1], 2015 im Nachgang zum sog. Sommer der Migration und aktuell mit Bezug auf Flucht aus der Ukraine verstärkte Aufmerksamkeit für die Situation von Geflüchteten und die damit verbundenen gesellschaftlichen Herausforderungen [2]. Diese punktuelle und auf das singuläre Ereignis fokussierte Perspektive verliert aus dem Blick, dass Deutschland bereits seit vielen Jahrzehnten ein Einwanderungsland ist und Migration als biografischer bzw. familiengeschichtlicher Prozess das Selbstverständnis einer substantiellen Bevölkerungsgruppe mitprägt.

Der Mikrozensus des Statistischen Bundesamtes aus dem Jahr 2020 [3] klassifizierte 26,7 % der Bevölkerung Deutschlands als „Personen mit Migrationshintergrund“^1^. Dazu kommen unter anderem noch internationale Studierende und Arbeitnehmende, Saisonarbeiter*innen, Menschen im Asylverfahren sowie undokumentierte Migrant*innen.

### Gesundheitssystem und Ausbildungsbedarf

Alle Menschen haben aus menschenrechtlicher Sicht dasselbe Recht auf medizinische Versorgung und bestmögliche Gesundheit [9]. Aus diesem zunächst abstrakten Recht auf Gesundheit ergeben sich konkrete, operationalisierbare Maßnahmen und Rahmenbedingungen für Gesundheitssysteme [10], die die Gesundheit aller in einem Land lebenden Menschen adressieren und nicht ausschließlich für Bürgerinnen und Bürger gelten [11].

Dem Gesundheitssystem obliegt daher die Aufgabe, seine Strukturen, Abläufe, Alltagspraktiken und Diskurse im Hinblick auf die Erfordernisse zur Sicherstellung dieser fundamentalen Rechte zu überprüfen und ggf. anzupassen [12]. Dieser Aufgabe wird das deutsche Gesundheitssystem hinsichtlich der Versorgung von Menschen mit Migrationsgeschichte bisher an vielen Stellen nur unzureichend gerecht: So ist für Geflüchtete während des Asylverfahrens oder mit einer Duldung der Zugang zu medizinischer Versorgung aus rechtlichen und bürokratischen Gründen eingeschränkt [13–16], während gleichzeitig die häufig prekären Lebensumstände nach der Ankunft in Deutschland und die unsichere Zukunftsperspektive das Krankheitsrisiko erhöhen [17, 18].^2^ Hinzu kommt, dass das deutsche Gesundheitssystem über keine systematischen Mechanismen zur Sicherstellung qualitativ hochwertiger Sprachmittlung verfügt [19, 20] und dass das Gesundheitspersonal häufig nicht ausreichend ausgebildet, durch strukturelle Rahmenbedingungen überfordert [21] oder unzureichend motiviert ist [22, 23], in sprachdiskordanten Situationen evidenzbasierte Lösungen zu finden. Zudem erfahren als migrantisch gelesene Patient*innen innerhalb des Gesundheitssystems immer wieder Diskriminierung und werden mit rassistischen Überzeugungen aufseiten des Gesundheitspersonals konfrontiert [14, 24]. Eine ausführliche Auseinandersetzung mit dieser Thematik findet sich im Beitrag von Kajikhina et al. in dieser Themenausgabe.

Während die Zuständigkeit für Veränderungen der strukturellen Bedingungen auf der Ebene des Politischen liegt, sind die Probleme innerhalb der klinischen Interaktion – sowie der Umgang mit sich aus den strukturellen Bedingungen ergebenden Eigenheiten in Bezug auf die Navigation im Gesundheitssystem – durch Verbesserungen der Ausbildung des Gesundheitspersonals beeinflussbar. Das Mandat für die Inhalte der Ausbildung liegt dahingehend bei den Gesundheitsberufen im Zuge ihrer Selbstverwaltung, sodass Defizite der Curricula zwar gesellschaftliche Muster und politische Diskurse widerspiegeln können, jedoch nicht ausschließlich politisch determiniert, sondern auch Ausdruck von Prioritätensetzungen innerhalb der Gesundheitsberufe sind.

Innerhalb der medizinischen Ausbildung gibt es in dieser Hinsicht substanzielle Defizite: So haben zwar die meisten in der Gesundheitsversorgung Tätigen im Arbeitsalltag regelmäßig Kontakt mit Patient*innen mit Migrationsgeschichte; ob ihr Studium und ihre Ausbildung sie für diese Kontakte gut vorbereitet haben, ist jedoch häufig von Zufall, individuellem Engagement einzelner Lehrender und von lokalen Bedingungen abhängig. So sind mit Migration in Zusammenhang stehende Themen – wie beispielsweise die professionelle Arbeit mit Dolmetschenden, Sensibilisierung für Diskriminierungsrisiken aufgrund von Rassismus, Vorurteilen und Stereotypen oder administrative Besonderheiten in Abhängigkeit vom Aufenthaltsstatus – bisher nicht longitudinal in den Pflichtcurricula des Medizinstudiums und anderer gesundheitsbezogener Studien- und Ausbildungsgänge verankert. Sie werden aktuell lediglich in fakultativen Lehrangeboten unterrichtet, die nicht alle Studierenden erreichen und zudem von Studienort zu Studienort in ihrem Umfang und ihrer inhaltlichen Ausrichtung variieren.

Vor diesem Hintergrund wurde im Verlauf des vergangenen Jahres das „Lehrnetzwerk Migration und Gesundheit“ gegründet, welches im Folgenden kurz vorgestellt werden soll.

## Bisherige Entwicklung und übergeordnete Ziele des Lehrnetzwerkes

Die erste veröffentlichte Lehrinitiative zum Thema Migration und Gesundheit in Deutschland startete 2005 mit dem Ziel, Aspekte der kulturellen Vielfalt in der Gesundheitsversorgung von Patient*innen mit Migrationshintergrund zu behandeln [25]. Solche Lehrangebote blieben bis 2015 in Deutschland allerdings die Ausnahme; erst im Kontext der Debatte um die Zuwanderung in den Jahren 2014 und 2015 wurde dann eine Reihe von neuen Wahlfächern und anderen Lehrformaten entwickelt, die die verschiedenen Facetten der Versorgung von Patient*innen mit eigener oder familiärer Migrationsgeschichte thematisieren [26–29].

Trotz dieser Zunahme von Kursen zu Migration und Gesundheit und zu interkultureller Kompetenz im Gesundheitswesen fehlt es bisher jedoch an systematischen Ansätzen zur Standardisierung von Inhalten auf der Grundlage von *Best Practices*, aktuellen theoretischen Konzepten und effektiven didaktischen Strategien; zudem mangelt es nach wie vor an der verbindlichen Integration dieser Themen in die Curricula [30]. Darüber hinaus besteht ein anhaltender Bedarf aufseiten der Studierenden, Fertigkeiten wie Diversitätskompetenz und interkulturelle Sensibilität oder die professionelle Arbeit mit Dolmetschenden zu lernen sowie Kompetenzen zu erwerben, um die Auswirkungen sozialer und politischer Determinanten der Gesundheit von Migrant*innen in der Versorgung zu adressieren [31].

Hierbei ist insbesondere wichtig, Lehransätze zu entwickeln und zu implementieren, die keine kulturalisierenden, behavioristischen oder rein biomedizinischen Perspektiven vertreten, da diese häufig in *Othering*^3^ münden und migrantische (oder als migrantisch gelesene) Bevölkerungsgruppen als Ausnahmefälle in einer ansonsten normierten Gesellschaft betrachten [32]. Vielmehr geht es darum, Gesundheitspersonal auf die zunehmende Diversität innerhalb von Gesellschaften vorzubereiten [33] und diese Diversität als Norm innerhalb einer von Globalität [34] und Mobilität [35] geprägten Welt zu betrachten. Hierin ergeben sich Synergien mit zahlreichen Initiativen zur Weiterentwicklung der Ausbildung verschiedener Gesundheitsberufe, so z. B. bei der verstärkten Integration globaler Gesundheitsthemen [30] oder der verstärkten Integration bevölkerungsmedizinischer Aspekte des öffentlichen Gesundheitswesens in die Curricula [36].

Inwiefern den oben beschriebenen Bedarfen oder Kompetenzdefiziten über Trainings- und Lehrangebote effektiv begegnet werden kann und welche Wirkung sie für die Versorgung entfalten, kann lediglich durch systematische Lehr- und Versorgungsforschung evaluiert werden. Begleitevaluationen bisheriger Lehrangebote weisen darauf hin, dass Studierende insbesondere von Lehrangeboten zur Kommunikation und Arbeit mit Dolmetschenden, zur Entwicklung interkultureller Sensibilität und zum Verständnis der psychosozialen Dimensionen der Gesundheit von Geflüchteten profitieren. Zudem zeichnet sich ab, dass Medizinstudierende hierbei das Lernen in multidisziplinären Teams als besonders bereichernd empfinden [26–29]. Für die Weiterentwicklung der Lehre in diesem Bereich wäre es auf diesen Ergebnissen aufbauend empfehlenswert, wenn Trainings- und Lehrangebote zukünftig stringenter und möglichst jenseits von Selbsteinschätzungen der Studierenden evaluiert würden und hierfür harmonisierte und standortübergreifende Instrumente eingesetzt würden.

Um einen Austausch zur Lehre im Themenfeld Migration und Gesundheit zu initiieren, haben sich ab Juni 2022 Vertreter*innen verschiedener Lehrinitiativen in einer Reihe von Videokonferenzen getroffen. Mittels Schneeballsystem wurden weitere Teilnehmende in den Prozess integriert. Die sich formierende Gruppe kam schließlich Ende März 2023 in Heidelberg zu einem 2‑tägigen Workshop zusammen, um das „Lehrnetzwerk Migration und Gesundheit“ zu gründen und ein Konzept für die weitere Zusammenarbeit zu entwickeln. Die Teilnehmenden des Workshops vertraten 11 Universitäten: Berlin, Bielefeld, Dresden, Freiburg, Gießen, Halle, Hamburg-Eppendorf, Heidelberg, Jena, Mainz und Würzburg. Im Verlauf des Workshops wurden die folgenden gemeinsamen Werte und Ziele für das Netzwerk formuliert: Übergeordnetes Ziel des Lehrnetzwerks Migration und Gesundheit ist es, eine menschenrechtsbasierte, diversitätssensible und Equity-orientierte Weiterentwicklung der Curricula des Medizinstudiums und anderer gesundheitsbezogener Studien- und Ausbildungsgänge zu fördern und die Entwicklung in Deutschland damit an internationale Standards anzunähern [37].

Das Lehrnetzwerk tritt dafür ein, dass das Studium und/oder die Ausbildung gemäß etablierten ethischen Standards zukünftiges Gesundheitspersonal darauf vorbereiten muss, eine diskriminierungsfreie Versorgung zu verwirklichen, gesellschaftliche Ungleichheiten und ihre gesundheitlichen Folgen zu adressieren und ein kritisches Bewusstsein für die gesellschaftliche Verantwortung von Gesundheitspersonal zu kultivieren.

## Handlungsbereiche des Lehrnetzwerkes

Zur Erreichung dieser übergeordneten Ziele soll das Netzwerk lehrbezogene Aktivitäten im Bereich „Migration und Gesundheit“ vernetzen und in 3 Handlungsbereichen bündeln:

1. Es soll eine Plattform geschaffen werden, über die sich Lehrende vernetzen können. Durch den Austausch von Lehrmaterialien und die gemeinsame Weiterentwicklung bestehender Lehrformate sollen die aktuell schon bewährten Konzepte weiter ausgearbeitet und zu Unterrichtshilfen ausformuliert werden.
2. Darauf aufbauend soll in Workshops kollaborativ ein Modellkurs erarbeitet werden, wobei die neuen Empfehlungen der Weltgesundheitsorganisation (WHO; [37]) zur Ausbildung von Gesundheitspersonal für den deutschen Kontext adaptiert und umgesetzt werden.
3. Parallel dazu wird das Lehrnetzwerk Strategien erarbeiten, um migrationsrelevante Inhalte longitudinal in Curricula zu implementieren. In Bezug auf das Medizinstudium wird geprüft, wie die Potenziale der neuen Approbationsordnung genutzt werden können, um die eingangs beschriebene Lücke zu füllen.

Diese Bestrebungen werden von Lehrforschung begleitet, die die Effekte bestehender und neu eingeführter Lehrveranstaltungen analysiert und eine Datengrundlage zur kontinuierlichen Weiterentwicklung schafft.

## Fazit und Ausblick

Das „Lehrnetzwerk Migration und Gesundheit“ ist die erste deutschsprachige Initiative, die versucht, migrationsrelevante Inhalte systematisch in der Aus- und Weiterbildung zu konsolidieren und weiterzuentwickeln. Damit zielt es darauf ab, eine Lücke in den Curricula der Gesundheitsberufe in Deutschland zu schließen. Bisher haben sich Lehrende aus 11 Universitäten dem Lehrnetzwerk angeschlossen und haben begonnen, eine Plattform für den Austausch von lehrbezogenen Aktivitäten im Bereich „Migration und Gesundheit“ zu etablieren. Dafür soll in einem nächsten Schritt ein Modellkurs zur Ausbildung von Gesundheitspersonal für den deutschsprachigen Kontext erstellt werden, der in der Praxis bereits bewährtes Material weiterentwickelt und didaktisch aufbereitet. Parallel dazu strebt das Lehrnetzwerk an, Wege zu finden, wie migrationsrelevante Inhalte longitudinal in die Curricula implementiert werden können, um die derzeitigen inhaltlichen Lücken zu schließen und die deutschen Curricula an internationale Standards anzunähern. Dafür laden wir herzlich zur aktiven Beteiligung am „Lehrnetzwerk Migration und Gesundheit“ ein (siehe Infobox).

### Infobox Einladung zur aktiven Beteiligung am „Lehrnetzwerk Migration und Gesundheit“

Das Lehrnetzwerk Migration und Gesundheit lädt alle Lehrenden im Bereich Migration und Gesundheit zur Mitarbeit ein, die sich an der kooperativen Weiterentwicklung der Lehre beteiligen möchten und die sich den oben genannten Werten verpflichtet fühlen.

Die Aktivitäten des Lehrnetzwerkes erfolgen in enger Zusammenarbeit mit dem Ausschuss Kulturelle Kompetenz und Global Health der Gesellschaft für Medizinische Ausbildung (GMA) sowie der Weltgesundheitsorganisation (WHO). Darüber hinaus sind auch weitere Arbeitsgruppen von Fachgesellschaften oder anderen Strukturen zur Zusammenarbeit herzlich eingeladen.

Wenn Sie als Lehrende*r dazu beitragen wollen, zukünftiges Gesundheitspersonal in ihrer Haltung und ihren Kompetenzen zu befähigen, eine gute Gesundheitsversorgung für alle Menschen in unserer Gesellschaft umzusetzen, dann laden wir Sie herzlich ein, Teil unseres Netzwerkes zu werden.

Sie erreichen uns unter: lehrnetzwerk@posteo.de.

## Footnotes

1 Das Konzept des „Migrationshintergrundes“ wird schon seit längerer Zeit kritisiert [4], da es Menschen mit sehr unterschiedlichen Migrationserfahrungen zu einer vermeintlichen Gruppe zusammenfasst, wodurch der Blick auf die Heterogenität der fraglichen Bevölkerung verstellt wird [5] und die Gefahr besteht, dass diese Kategorisierung *Othering*, rassistischer Diskriminierung und anderen Ausschlüssen Vorschub leistet [4, 6]. Hinzu kommt, dass die Forschung der letzten Jahre zunehmend gezeigt hat, dass unterschiedliche Migrationserfahrungen sich jeweils unterschiedlich auf gesundheitliche Risiken auswirken und der epidemiologische Nutzen dieser Kategorie zweifelhaft ist [7]. Das Robert Koch-Institut empfiehlt daher, zukünftig auf reduktionistische Klassifikationen zu verzichten und stattdessen die Migrationserfahrung strukturierende biografische, soziale, migrationsbezogene und strukturelle Faktoren separat zu erheben [8]. Im Folgenden sprechen wir von *Personen mit eigener oder familiärer Migrationsgeschichte*, um auf Personen zu verweisen, deren Biografie durch Migrationserfahrung mitgeprägt ist.

2 Für eine ausführliche Auseinandersetzung mit den *Determinanten der Gesundheit und medizinischen Versorgung von Asylsuchenden* sowie den Auswirkungen dieser Determinanten auf den Gesundheitszustand siehe auch den Beitrag von Führer in dieser Themenausgabe.

3 Der Begriff Othering (aus dem engl. „other“ = „andersartig“) beschreibt die Distanzierung und Differenzierung zu anderen Gruppen, um seine eigene „Normalität“ zu bestätigen. Es bedeutet auch, dass Menschen oder Gruppen negative Eigenschaften zugeschrieben werden, die sie von der eigenen sozialen Gruppe unterscheiden, die als normativer Standard wahrgenommen wird.
